# Nutritional and physiological limitations shape the radiation-use efficiency response to legume proportion in C_4_ grass–legume mixtures

**DOI:** 10.1093/aobpla/plaf036

**Published:** 2025-06-26

**Authors:** Nicolas Caram, Lynn E Sollenberger, Marcelo O Wallau, Jose C B Dubeux, Chris H Wilson

**Affiliations:** Agronomy Department, University of Florida, Gainesville, FL 32611, United States; Departmento de Producción Animal y Pasturas, Facultad de Agronomía, Universidad de la República, Paysandú 60000, Uruguay; Agronomy Department, University of Florida, Gainesville, FL 32611, United States; Agronomy Department, University of Florida, Gainesville, FL 32611, United States; North Florida Research and Education Center, University of Florida, Marianna, FL 32446, United States; Agronomy Department, University of Florida, Gainesville, FL 32611, United States; Plants, Ecosystems & Climate

**Keywords:** bahiagrass, grasslands, legume proportion, resource-use efficiency, rhizoma peanut

## Abstract

Legume introduction is effective for boosting primary productivity in C_4_-grass-dominated subtropical and tropical grasslands by overcoming nitrogen (N) limitation and consequently improving radiation-use efficiency (RUE), a key metric underlying plant production. However, an excessive proportion of C_3_ legumes may negatively affect RUE, especially in warm climates. We assessed the relationship between aboveground RUE and legume proportion of *Paspalum notatum* Flügge (C_4_ grass) mixtures with a tropical legume (*Arachis glabrata* Benth.; 0%–80%) under different defoliation and N fertilizer treatments in two studies over 3 years in Florida, USA. Linking the field data to a conceptual model, RUE was optimized at 26%–30% legume proportion across studies and years. When pastures were N-fertilized, RUE plateaued at 26% legume (0.60 g MJ^−1^) and linearly decreased with higher legume proportions. When pastures were unfertilized, RUE showed a quadratic relationship with legume proportion, being maximized at 30% legume (1.10 g MJ^−1^), overyielding the RUE in only-grass and legume-dominated sites by 110% and 86%, respectively. These responses suggest that RUE is N-limited when legume is below 30% in unfertilized canopies and is physiologically limited when legume is above 30% due to replacement of the C_4_ grass with a C_3_ legume. These findings provide a robust rationale to target low-to-moderate legume proportions in tropical grasslands for optimizing production and other ecosystem services. We empirically demonstrated that optimum legume proportion is ∼30% in a C_4_-grass-based tropical grassland compared with previous observations of ≥40% in C_3_-grass-based temperate grasslands, relevant insights for the design and maintenance of grassland ecosystems.

## Teaser

Linking field data of a warm-climate C4 grass-legume mixture to a conceptual model, we found that the radiation use efficiency, a proxy of primary productivity, was optimized at 30% legume proportion across different studies and years. When these warm-climate mixture were N-fertilized, radiation use efficiency plateaued at 26% legume and then linearly decreased with higher legume proportions. However, when these pastures were unfertilized, the radiation use efficiency quadratically increased with legume proportion, up to an optimum at 30% legume. Beyond this proportion, it decreases following the same pattern as the N-fertilized pasture. These responses suggest radiation use efficiency, and likely primary productivity, are N-limited when legume is below 30% in unfertilized canopies and are physiologically-limited when legume is above 30% due to replacement of the C4 grass with a C3 legume. These findings suggest that low to moderate legume proportions can maximize production and other ecosystem services in warm-climates.

## Introduction

Global grazing lands occupy about 37% of terrestrial land area (O’Mara 2012), store a similar proportion of the global soil carbon (Bardgett et al. 2021, Bai and Cotrufo 2022), and play critical roles in our global agricultural systems (O’Mara 2012, Sollenberger et al. 2019). However, with rising pressures from global population and economic expansion and worsening climate change, there is a need to sustainably intensify the use of grasslands (Soussana and Lemaire 2014, Sollenberger et al. 2019). Although a variety of practices contribute to the sustainable intensification of grasslands, legume introduction is one of the most promising for boosting productivity, enhancing soil carbon, and reducing fertilizer inputs and associated emissions, particularly for warm-season, C_4_-grass-dominated subtropical and tropical grasslands (Muir et al. 2011, Sollenberger et al. 2019). Despite the widely recognized value of legume incorporation in both ecological (e.g. Tilman et al. 2001, Fornara and Tilman 2009) and agronomic systems (e.g. Helgadóttir et al. 2018, Jaramillo et al. 2021, Sollenberger and Dubeux 2022, Caram et al. 2025a), we still lack a strong theoretical and practical basis for identifying a target legume proportion to optimize productivity and other ecosystem services and likewise for prescribing management interventions to move systems towards that optimum. Here, we discuss and apply a key ecophysiological concept, the so-called radiation-use efficiency (RUE, Monteith 1972, 1977), to the case of identifying optimum legume proportion in a subtropical pasture system, using data from two field studies linked to a conceptual model.

RUE is an integrative metric, defined as the biomass produced per unit of solar radiation intercepted or absorbed by the canopy in a given period of time (Monteith 1972, 1977). More specifically, RUE can be defined as the net primary production per absorbed or intercepted photosynthetically active radiation (iPAR) and can be reported relative to the proportion of carbon allocated to different organs (i.e. aboveground RUE, total RUE, Bélanger et al. 1994). Measurement of RUE enables greater understanding of species growth and efficiency under different practices, environments, and theoretical scenarios associated with climate change (Sinclair and Muchow 1999, Stöckle and Kemanian 2009, Peng et al. 2020). Thus, identifying management practices that optimize RUE is critical for achieving sustainable intensification of grassland ecosystems (Soussana and Lemaire 2014). In this regard, while incorporating legumes alongside C_4_ grasses can help overcome the N limitation on primary productivity (Tilman et al. 2001, Fornara and Tilman 2009, Kohmann et al. 2018, Garcia et al. 2021, Jaramillo et al. 2021), empirical evidence also indicates that primary productivity, and likely RUE, decreases when legume proportions are too high (Kohmann et al. 2022, Caram et al. 2025b).

Higher proportions of legume might lead to reduced RUE because species of different photosynthetic pathways and functional groups vary in RUE (Sinclair and Horie 1989, Faurie et al. 1996, Sinclair and Muchow 1999, Stöckle and Kemanian 2009, Druille et al. 2019). For example, C_4_ species have greater RUE than C_3_ species, ranging between 1.0 and 2.0 g dry matter (DM) MJ^−1^ iPAR under field conditions (Sinclair and Muchow 1999, Kiniry et al. 2007, Stöckle and Kemanian 2009), and above 2.0 g MJ^−1^ iPAR under conditions of abundant nitrogen (N) and light (e.g. Bélanger et al. 1994, Cruz 1997, Gómez et al. 2013). In general, C_3_ grass monocultures average 1.4 g MJ^−1^ iPAR (Monteith 1977, Stöckle and Kemanian 2009). When in mixture with legumes, RUE ranges from 0.9 to 1.4 g MJ^−1^ iPAR according to N and defoliation treatments (Ojeda et al. 2018, Grigera and Oesterheld 2021). The lowest RUE values are seen in native grassland ecosystems, i.e. rangelands, where RUE ranges from 0.2 to 1.0 g MJ^−1^ iPAR (Piñeiro et al. 2006), likely associated with N limitation (Boggiano et al. 2001). Therefore, one of the potential reasons for the reduced RUE with higher legume proportions is the generally lower inherent RUE of legumes compared with C_4_ grasses (Sinclair and Muchow 1999, Stöckle and Kemanian 2009), an effect likely exacerbated under high temperatures in warm climates.

**Figure 1.**
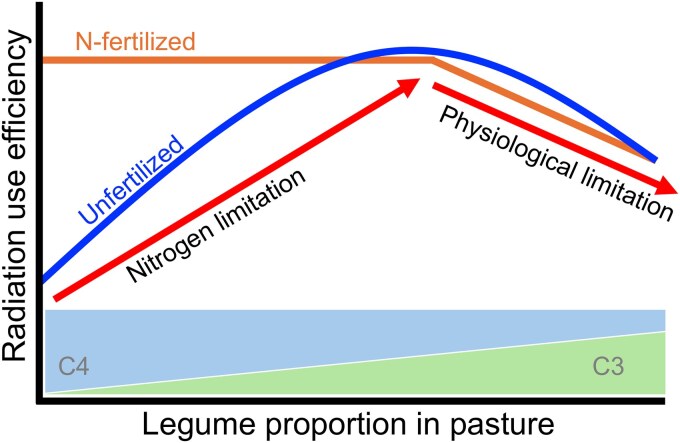
Hypothetical conceptual model of the relationship between RUE and legume proportion in tropical environments, including potential factors limiting RUE along the legume gradient.

We synthesize this evidence with a hypothetical ecophysiological model relating RUE with legume proportion (Fig. 1). According to this conceptual model, there is a hypothetical optimum legume proportion in unfertilized pastures where RUE is maximized, resulting from a decline in resource limitation with higher proportions of legumes (e.g. N through biological N fixation and other resources facilitation; Barry et al. 2019) trading off with an increase in physiological limitations (i.e. lower RUE; Fig. 1). In N-fertilized pastures, the N limitation is partially or fully overcome, leading to a decrease in RUE as legume proportion increases (i.e. physiological limitation, e.g. Caram et al. 2025a). Alternatively, RUE may initially plateau as the legume helps overcome not only N but also other limiting resources through facilitation and niche partitioning (Barry et al. 2019) and then decreases with high legume proportions.

From a management practice perspective, the identification of the optimum legume proportion would potentially overcome the N limitation without compromising RUE and therefore maximize primary productivity. However, this potential optimum is not merely determined by genetics (species-inherent), as RUE is highly sensitive to environmental conditions and management-related factors, including canopy architecture, temperature, photoperiod, and most importantly soil moisture and N nutrition (Faurie et al. 1996, Sinclair and Muchow 1999). Indeed, legumes often have more horizontal leaves than grasses (e.g. Husse et al. 2016). All else equal, species with more horizontal leaves, i.e. greater light extinction coefficient (*k*), have lower RUE associated with a poor distribution of light throughout the canopy, while upper leaves light saturate (Sinclair and Horie 1989). However, these species with more horizontal leaves can intercept greater solar radiation at similar leaf area index (LAI), reaching earlier the ‘optimum’ LAI, i.e. the stage where 90%–95% of incident light is intercepted and net biomass accumulation is maximized (Brougham 1958). Thus, grass–legume mixtures of different canopy architectures might respond differently in photosynthesis rate and net biomass accumulation (Monteith 1972, Parsons et al. 1983) and botanical composition (Tilman 1985) to specific defoliation/grazing management practices. In addition, canopy attributes and responses and N nutrition can also interact with each other, often non-linearly, adding complexity to identification of factors limiting RUE and management practices for maximizing pasture RUE and, consequently, primary productivity. Thus, photosynthetic pathway (C_3_ vs. C_4_), plant functional group (grass vs. legume), and soil N availability can have conflicting effects on RUE, especially in warm-climate grasslands where N is typically the main limiting resource defining primary productivity (Fig. 1).

Therefore, identifying potential optimum legume proportions maximizing RUE in any given environment and management context requires a quantitative assessment of RUE in response to a legume gradient while considering different resource availability scenarios, particularly soil N. Here, we assess the effects of legume introduction on aboveground RUE (i.e. biomass production) with the overall goal of identifying an optimal legume proportion for maximizing RUE in a warm-climate grassland under different N nutrition levels, thereby testing the hypothetical conceptual model proposed in Fig. 1. Our specific objectives were to (i) quantify canopy characteristics and relationships among canopy height, light interception, and LAI of a C_4_ grass (bahiagrass; *Paspalum notatum* Flügge) monoculture and in mixture with a C_3_ perennial legume (rhizoma peanut; *Arachis glabrata* Benth.) under different defoliation and N fertilization regimes, during 3 years in Florida, USA, and (ii) assess the qualitative relationship of legume introduction (grass vs. grass–legume) and the quantitative relationship of relative proportion of legume and aboveground RUE, using quantile regressions of linear models (first- and second-order regression) and non-linear models (plateau-linear and plateau-quadratic regressions), to test different shapes in the RUE response to legume proportion.

## Materials and methods

### Site

Two observational studies were carried out at the Beef Research Unit of the University of Florida, Gainesville, FL (29.74 N, 82.27 W), during 2021, 2022, and 2023. The areas for both experiments were in close proximity to each other (Supplementary Appendix S1). The site areas are dominated by Chipley sand soils that are rapidly permeable. Study 1 was conducted in small plots under clipping defoliation management from May to October 2021 and 2022, while Study 2 was conducted in grazed pastures from June to October 2022 and 2023.

### Design

Both observational studies were conducted in ‘Pensacola’ bahiagrass monocultures and mixtures with ‘Ecoturf’ (Study 1) or ‘Florigraze’ (Study 2) rhizoma peanut. In Study 1, 3-m^2^ sites (3 m × 1 m) were selected in four larger plots of bahiagrass monocultures and mixtures (i.e. ‘main plots’, *n* = 8), hereafter referred to as ‘canopy types’. The Ecoturf rhizoma peanut was established in 2016 by planting its rhizomes uniformly throughout the plot area, previously prepared by disking and herbicide application, while concurrently broadcast seeding Pensacola bahiagrass. The realized legume proportion during 2022–2023 ranged between 1% and 80%. The 3-m^2^ sites were divided into 1-m^2^ ‘sub-plots’ and manipulated according to defoliation and N fertilization: (i) undefoliated receiving no N fertilizer (only clipped by the end of the growing season), (ii) clipped to 5 cm when LAI was >3 and receiving no N fertilizer, and (iii) clipped to 5 cm when LAI was >3 and fertilized immediately after harvest with 20 kg N ha^−1^ as ammonium nitrate (*n* = 24). These 24 sites were repeatedly assessed during the growing seasons of 2021 and 2022. The LAI > 3 threshold was chosen in an attempt to competitively balance the bahiagrass component against competition from the lower-growing, decumbent Ecoturf rhizoma peanut cultivar. Our rationale was that a longer regrowth interval, as indexed by an LAI of ∼3, would allow the grass to partially shade out the legume, resulting in an average regrowth interval of 40 days.

In Study 2, 1-m^2^ sites of bahiagrass monoculture receiving no N fertilization and of bahiagrass–rhizoma peanut mixtures were selected in eight pastures of 0.5 ha each. Three sites were selected in each of two 0.5-ha naturalized bahiagrass monoculture pastures (*n* = 6; 0% legume). Three sites were selected in each of six 0.5-ha grass–legume mixture pastures (*n* = 18), varying in legume proportion, totalling 24 1-m^2^ sites simultaneously assessed during 2022 and 2023. These six pastures were initially established as rhizoma peanut monocultures in 1983 (Ortega-S. et al. 1992) and afterwards were colonized by bahiagrass creating mixtures with different proportions of each species (15%–60% legume; Caram et al. 2025a). To prevent grazing in these sites, three exclusion cages were placed in each of the eight pastures (*n* = 24). Caged sites were clipped every 4 weeks, and once clipped, new sites close to the previous ones were selected and then caged. The eight pastures were continuously stocked during the growing seasons of 2022 and 2023, under the same grazing intensity, defined based on herbage allowance (Sollenberger et al. 2005), which relates the biomass offer, in kg DM, and the animal live weight.

### Sampling procedures and measurements

Light interception [fractional photosynthetically active radiation (fPAR)] and LAI were estimated weekly in each 1-m^2^ site of Study 1 using a LiCOR LAI-2200C. Simultaneously, the compressed canopy height was measured in each site using an electronic rising plate meter (Platemeters, NZ). The sites were clipped, except for the undefoliated control, when canopies achieved an LAI of >3, resulting in four clipping events in 2021 (23 June, 2 August, 7 September, and 25 October) and five in 2022 (31 May, 6 July, 1 August, 15 September, and 26 October). The undefoliated control was clipped on the final sampling day each year, 25 October 2021 and 26 October 2022. The proportion of bahiagrass and rhizoma peanut in total biomass was measured in each site in June 2021 and July 2022. Species were hand separated and dried at 60°C for 72 h to determine dry mass and legume proportion (%) in the canopy.

For Study 2, the LAI and canopy light interception were estimated weekly in the centre of each caged site using a LiCOR LAI-2200C. Simultaneously, the compressed canopy height was measured using a rising plate meter (Rayburn and Rayburn 1998). In the centre of each cage, an area of 0.25 m^2^ was clipped to 1 cm after a 4-week period. After clipping, new 1-m^2^ sites were selected and caged near the previous ones and then an area of 0.25 m^2^ was clipped after a 4-week period. In total, there were four sampling events during the growing season of 2022 (21 July, 19 August, 16 September, and 14 October) and 2023 (6 July, 3 August, 1 September, and 29 September). Species were hand separated and dried at 60°C for 72 h to determine dry mass and legume proportion (%). All clipped samples were dried, weighed, ground to pass a 1-mm stainless steel screen using a Wiley mill (Model 4 Thomas-Wiley Laboratory Mill, Thomas Scientific), and analysed for N concentration (% N) as an indication of the N nutritional status of canopies.

The paired data of light interception and LAI were used to fit the Beer–Lambert law equation (Monsi and Saeki 2005), as follows:


(1) $$\mathrm{fPAR}=1-e^{-k*LAI}$$

where the light interception (i.e. fPAR) exponentially increases in response to the LAI and the *k* determines the shape of the curve. Canopies with more horizontal leaves will have greater *k* and may compromise the RUE, as upper leaves may light saturate (Sinclair and Horie 1989). Using the compressed canopy height paired data, it is possible to assess the canopy density (LAI cm^−1^), and compressed canopy height needed to intercept a targeted fraction of the PAR.

Daily solar radiation (MJ m^2^) was acquired from the Florida Automated Weather Network (https://fawn.ifas.ufl.edu) for Alachua County (located 15.4 km from the experimental area) and then converted to PAR using a factor of 0.48. The aboveground RUE of a given canopy was estimated by solving the following equation:


(2) $$\frac{\Delta \mathrm{biomass}}{\Delta \;\mathrm{time}}=\mathrm{PAR}*\mathrm{fPAR}*\mathrm{RUE}$$

where Δ biomass/Δ time is the aboveground biomass accumulation in a certain period of time, PAR is the photosynthetically active radiation, estimated as a fraction (0.48) of the solar radiation (MJ m^2^), fPAR is the fraction of the PAR intercepted by the canopy, and aboveground RUE is the radiation-use efficiency (g MJ^−1^). The cumulative iPAR (PAR * fPAR) for a given clipping event was estimated as the sum of weekly assessments of fPAR and cumulative PAR during that week. Therefore, aboveground RUE was estimated as:


(3) $$\mathrm{RUE}=\frac{\left(\;\frac{\Delta \mathrm{biomass}}{\Delta \;\mathrm{time}}\right)}{\sum \;(\mathrm{PAR}*\mathrm{fPAR})}$$

This equation does not consider potential senescence and herbivory losses, which were likely to be negligible, as most clipping events were within a 4-week period (except for the undefoliated control in Study 1), and sites in grazed pastures were caged to avoid grazing events.

The biomass accumulated in each clipping event in Study 1 (four in 2021 and five in 2022) was considered as the DM harvested above 5 cm. Potential biomass produced below 5 cm was not considered. The biomass accumulated in each site in Study 2 was estimated as the difference between final and initial biomass for each clipping event (four in 2022 and 2023). Initial biomass and final biomass were estimated using the double-sampling technique (Haydock and Shaw 1975). Equations of disk height and biomass for each pasture at initial and final clippings were built using direct measurements of biomass and compressed canopy height with the rising plate meter. The equations were then used to predict the biomass in the caged site. The coefficient of determination averaged 83.2% (±8.6%) during 2022 and 85.3% (±9.8%) during 2023.

### Data analysis

To address Objective 1 of quantifying canopy characteristics of bahiagrass monocultures compared with the grass–legume mixtures, as determinants of biomass accumulation, we tested differences in *k*, i.e. the light extinction coefficient, between canopy types and manipulation treatment (control, clipped 0N, and clipped +N) fitting the non-linear Beer–Lambert law (1) within mixed models, using the *nlme* function of the ‘nlme’ package (Pinheiro et al. 2021). The response variable was the light interception (fPAR) relative to the canopy LAI. For Study 1, we tested differences in *k* between canopy types within manipulation treatments. For Study 2, we tested differences in *k* between canopy types. Canopy types and treatments, when present, were included as fixed effects, while plots and sub-plots within plots (Study 1) and sites within pastures (Study 2) were included as random effects.

In addition, we tested differences in canopy density, regressing LAI against compressed canopy height, between canopy types within manipulation treatments, when present, for each study, fitting linear mixed models using the *lme* function of the ‘nlme’ package (Pinheiro et al. 2021). For both studies, compressed canopy height, pasture, treatment, and their interactions were included as fixed effects, and similarly, plots and sub-plots within plots (Study 1) and sites within pastures (Study 2) were included as a random effect. The slope of the LAI on canopy height, i.e. canopy density (LAI cm^−1^), was tested among treatments and between canopy types using the *emtrends* function of the ‘emmeans’ package.

To address Objective 2 of investigating the relationship between legume presence and proportion and aboveground RUE under different N and defoliation treatments, we first studied the qualitative relationship of canopy types–treatments and aboveground RUE, performing linear mixed models using *lme* (Pinheiro et al. 2021). We included canopy types, treatment (only for Study 2), year, and the interactions as fixed effects and site as a random effect. The assumptions of residuals for all models were assessed graphically (Kozak and Piepho 2018).

We then explored the relationship between legume proportion and aboveground RUE for both studies using regression analysis. We studied this association for each study pooling Year 1 and Year 2 data (overall response) and then individually for each year. In addition, we tested the median response of RUE and legume proportion (50th quantile), the frontier response of RUE and legume proportion where, theoretically, the association is only limited by legume proportion (95th quantile; Cade et al. 1999, Cade and Noon 2003), and the limited response of RUE and legume proportion where resources other than legume proportion limit the shape of the relationship (10th quantile).

To distinguish the ‘best’ candidate model describing these relationships and therefore estimate the potential optimal legume range maximizing RUE, we performed linear and non-linear models. The candidate models were first- and second-order linear regressions and plateau-linear and plateau-quadratic non-linear regressions. These four candidate models were performed for each study, for the Year 1 and Year 2 pooled data (overall response), for each year within studies, and for each quantile (10th, 50th, and 95th), and for growth seasons during both years. The ‘best’ candidate quantile regression model in each case defining the shape of the relationship was selected based on the lowest Akaike information criteria (AIC), the corrected AIC (AICc; Burnham and Anderson 2002), and root mean square error (RMSE) out of sample. In this case, we performed the leave-one-out cross-validation technique for model evaluation through RMSE. Each model was trained with *n*-1 observations and then tested with the observation that was left out. This process was repeated for all observations, where the overall RMSE corresponded to the average of RSMEs estimated in each iteration.

## Results

### Canopy attributes

**Figure 2.**
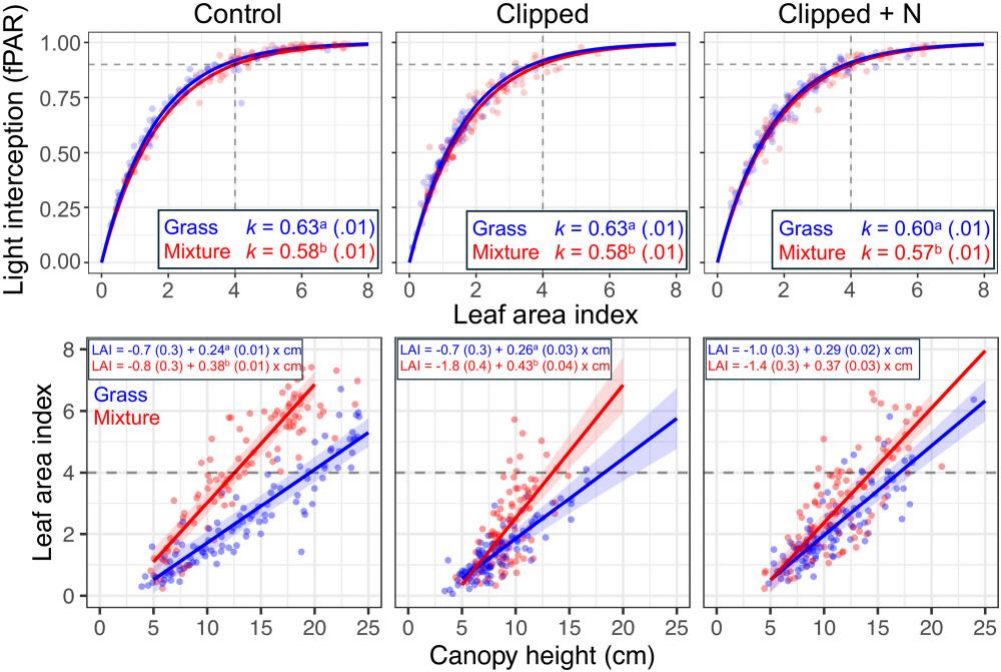
Canopy attributes in the clipping study (Study 1). The upper panel shows the light interception response to LAI (as an fPAR) for canopy types and defoliation and N-fertilization manipulation. The horizontal and vertical dashed grey lines denote the 90% light interception and the corresponding LAI, respectively. The lower panel shows the canopy density for canopy types and each defoliation and N-fertilization manipulation, expressed as units of LAI per unit of compressed canopy height (LAI cm^−1^). The horizontal dashed grey lines denote the LAI where ∼90% of incident light is intercepted. Different letters following means (SE) indicate differences between canopy types within manipulation treatments at *P* < 0.01.

**Figure 3.**
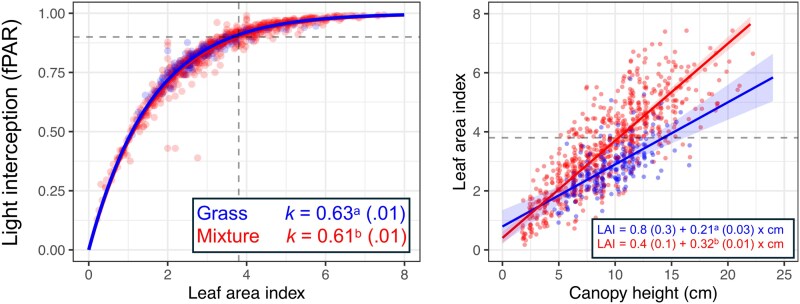
Canopy attributes in the grazing study (Study 2). The left panel shows light interception response to LAI (as an fPAR) for each canopy type. The right panel shows the canopy density for each canopy type, expressed as units of LAI per unit of compressed canopy height (LAI cm^−1^). Different letters following means (SE) indicate differences between canopy types at *P* < 0.01.

Canopies from both studies and of both types intercepted 90% of PAR at LAI 3.8–4.1 (Figs 2 and 3). In all cases, the light extinction coefficient (*k*) was statistically greater in bahiagrass monocultures compared with the mixtures. Despite having similar LAI intercepting a target ‘optimum’ 90% of PAR, the canopy density, expressed as LAI units per compressed canopy cm (LAI cm^−1^), differed between canopy types (Figs 2 and 3). Except for the N-fertilized, clipped treatment in Study 1, mixtures showed a 50%–60% denser canopy than the bahiagrass monocultures. As a result, the same target LAI is achieved with shorter compressed canopy heights in mixtures than in bahiagrass monocultures.

### Aboveground radiation-use efficiency

**Figure 4.**
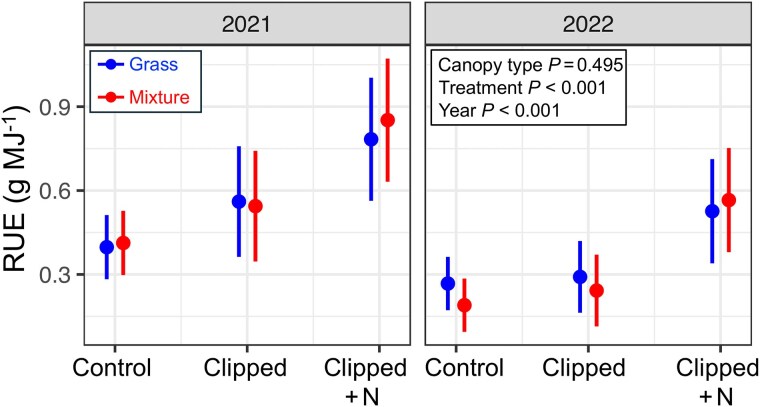
Aboveground RUE in response to manipulation treatments (undefoliated control, unfertilized clipped, and clipped +N) and canopy type (grass and mixture) for the clipping study (Study 1) during 2021 and 2022. The *P*-values correspond to the significance of main effects. Vertical lines indicate ±95% confidence intervals.

RUE was greatest in clipped N-fertilized sites (0.68 ± 0.05 g MJ^−1^), intermediate in unfertilized, clipped sites (0.41 ± 0.05 g MJ^−1^), and least in undefoliated control sites (0.32 ± 0.05 g MJ^−1^; Fig. 4, *P* < 0.001). By contrast, RUE differed minimally among grass–legume mixtures and bahiagrass monocultures (0.46 vs. 0.47 ± 0.05 g MJ^−1^; *P* = 0.495). However, for Study 2, the RUE was statistically greater under grass–legume mixtures than under bahiagrass monocultures (0.97 vs. 0.43 ± 0.12 g MJ^−1^; *P* < 0.001).

**Figure 5.**
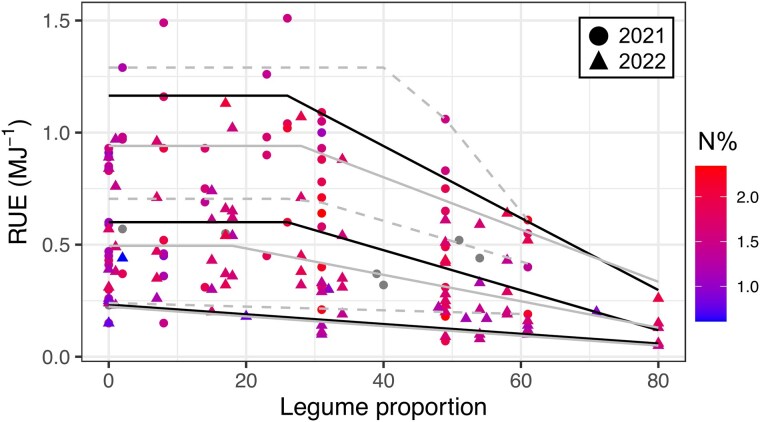
Relationship between aboveground RUE and legume proportion in the clipping study (Study 1) during 2021 and 2022. The N% indicates the N concentration of the aboveground biomass. The three quantile lines report the association between median RUE and legume proportion (50th quantile), unlimited response (95th quantile), and limited response (10th quantile). Solid (2021) and dashed (2022) grey lines indicate the best fitted regression for each year. Missing values for N% are in grey.

**Figure 6.**
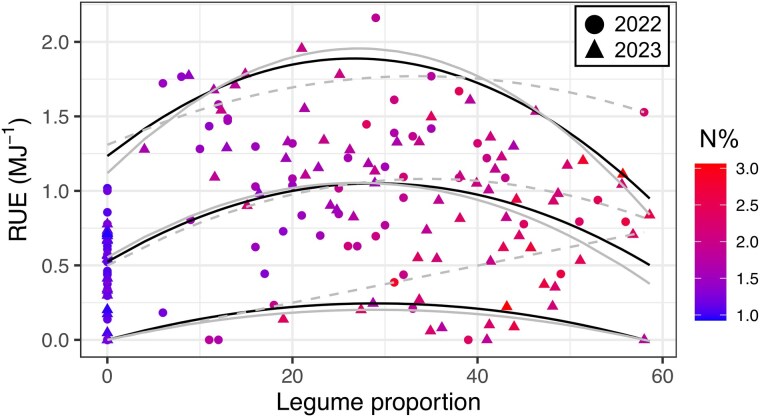
Relationship between aboveground RUE and legume proportion in the grazing study (Study 2) during 2022 and 2023. The N% indicates the N concentration of the aboveground biomass. The three quantile lines report the association between median RUE and legume proportion (50th quantile), unlimited response (95th quantile), and limited response (10th quantile). Solid (2021) and dashed (2022) grey lines indicate the best fitted regression for each year.

Quantitatively exploring the relationship between RUE and legume proportion in pasture, we consistently found that aboveground RUE decreased with legume proportions greater than ∼30% (Figs 5 and 6). The median (50th quantile) of RUE in Study 1 showed an apparent linear plateau response to legume proportion in pasture (overall response; Supplementary Appendix S2). RUE plateaued with legume proportion up to 26% (averaging 0.60 g MJ^−1^ across treatments); beyond this critical value, it decreased linearly at rate of −0.009 g MJ^−1^ per unit increase in legume. This pattern was also observed across growth seasons, where RUE decreased beyond 25% legume from late spring to mid-summer (Supplementary Appendix S3). Hence, increases from 30% to 50% in legume proportion are associated with decreases in RUE of 0.18 g MJ^−1^. According to the best model for 2021, RUE plateaued at 0.70 g MJ^−1^ until 30% legume and then decreased at a rate of −0.009 g MJ^−1^ per percentage unit increase of legume. Similarly, for 2022, the RUE plateaued at 0.50 g MJ^−1^ until 18% legume and then decreased at a rate of −0.006 g MJ^−1^ per percentage unit increase of legume.

The frontier response, i.e. 95th quantile, indicated a similar pattern to the median response (Fig. 5; Supplementary Appendix S2). Averaging both years, RUE plateaued at 1.17 g MJ^−1^ until 26% legume; above this level, it decreased at 0.016 g MJ^−1^ per percentage unit of legume. This suggests that under non-limiting conditions, where theoretically legume is the only limiting factor, RUE also decreased beyond a critical proportion of 26% legume, at greater rates compared with the median response (−0.016 vs. −0.009 g MJ^−1^, respectively). For 2021 and 2022, RUE plateaued at 1.29 and 0.94 g MJ^−1^ until 43% and 28% legume, respectively. Beyond this critical value, it decreased at 0.038 and 0.012 g MJ^−1^ per percentage unit of legume, for 2021 and 2022, respectively. On average for both years, the 10th quantile, i.e. when RUE is limited by factors other than legume proportion, RUE linearly decreased from 0.23 g MJ^−1^ at 0% legume at a rate of −0.002 g MJ^−1^ per one-unit increase in legume.

For Study 2, on average for 2022 and 2023 and for individual years, we found a quadratic relationship between the median and unlimited RUE (50th and 95th quantile) and legume proportion (Fig. 6; Supplementary Appendix S4). The RUE averaged 0.53 g MJ^−1^ at 0% legume (i.e. intercept of the regression; 0.45 and 0.57 g MJ^−1^ for 2022 and 2023, respectively), similar to the intercept in Study 1. On average, RUE peaked at 31% legume in pasture (33 and 28% for 2022 and 2023, respectively) and then quadratically decreased. The RUE at the optimum legume proportion of 31% legume was 1.10 g MJ^−1^, overyielding the 0% legume and 60% legume by 110% and 86%, respectively. A similar pattern was also detected across growth seasons, where RUE showed a quadratic relationship with legume proportion from late spring to mid-summer, peaking at 32% legume (Supplementary Appendix S5). Under unlimited conditions during the growing season, i.e. 95th quantile, the RUE averaged 1.23 g MJ^−1^ at 0% legume, similar to the 95th quantile for Study 1% and 116% greater than the median response. The 95th quantile RUE peaked at 27% legume (33% and 27% for 2022 and 2023, respectively) and then decreased at a greater rate than for the median. When limiting resources included those not associated with legume proportion, i.e. 10th quantile, the RUE quadratically increased with legume proportion until 30%.

Based on our findings, we propose that an appropriate ‘optimum’ legume inclusion in C_4_ grass pasture where RUE is maximized, integrated across N levels and environmental factors, occurs at ∼30% legume. The RUE of C_4_ grass pastures containing a low proportion or no legumes (0% to 30%) is limited by a nutritional N deficiency, which is overcome with N fertilization (as shown by the difference in shape response in Figs 5 and 6). At an ‘optimum’ legume proportion of ∼30%, the RUE is maximized for canopies receiving no N (Fig. 6). However, it decreases beyond 30% (Figs 5 and 6), suggesting that as proportion of C_3_ legumes exceeds 30%, RUE in C_4_ grass pastures will decrease, likely associated with a physiological limitation of the RUE.

## Discussion

The current demand to achieve sustainable intensification in agricultural systems requires the identification of practices optimizing resource-use efficiency (Soussana and Lemaire 2014). In this context, RUE is a uniquely integrative metric to assess management practices, encompassing the net response to environmental conditions and management practices. In the case of subtropical pastures, previous work has strongly supported that introducing legumes into only-grass systems enhances primary and secondary production, and other ecosystem services (Jensen et al. 2012, Sollenberger et al. 2019, Sollenberger and Dubeux 2022). An important next step for sustainable management of legumes in C_4_ grass pastures is to identify an optimal range of legume proportion, something which previous literature has not convincingly accomplished (Sollenberger et al. 2019). In this study, we found that RUE maximizes at ∼30% legume proportion across two distinct studies within our study system, adding robustness to the findings. Consistent with the conceptual model introduced in Fig. 1, we posit that ∼30% legume proportion substantially alleviates N limitation, without the need of N fertilizer inputs, while the RUE is not compromised due to an excessive proportion of C_3_ species in this C_4_ grassland. Thus, our study suggests that practical managements should target moderate legume proportions to maximize primary productivity of warm-climate grassland ecosystems. On the contrary, the proposed ecophysiological conceptual model could be tested in other warm-climate regions including different tropical grass and legume species, which highlights the contribution of this research and its generalizable practical and theoretical findings.

While qualitative comparison of canopy types does not provide insight into the relationships between RUE and legume proportion, quantitative analyses testing linear and non-linear responses between RUE and legume proportion consistently showed a decrease in aboveground RUE when legume proportion was above ∼30% (Figs 5 and 6). This response occurred in both cultivars having similar aboveground biomass production (Mullenix et al. 2016), suggesting that the difference in the shape of the response between studies was likely due to N nutrition level at low legume proportion (Fig. 1). In Study 1, the N concentration did not vary along the plateau of the median and 95th quantile response (Fig. 5), maintaining the RUE along the initial legume gradient in the clipped, N-fertilized treatment. In Study 2, the N concentration increased with initial increases in legume proportion (Fig. 6), likely explaining the increases in 50th and 95th RUE until ∼30% legume. Beyond this point and in both studies and for the 2 years and different growth seasons during the year, the RUE declined either linearly or quadratically in spite of the N concentration.

Here, we infer that this reduction in aboveground RUE is a physiological limitation of the canopy (Fig. 1). In other words, RUE is compromised with an excessive proportion of C_3_ species (i.e. legume) in this warm-climate environment. Thus, RUE, and therefore, primary production maximize at proportions of about 30% legume in these C_4_-grass-based pastures. Interestingly, a higher optimum proportion has been identified for temperate grass–legume mixtures (e.g. Nyfeler et al. 2009, Lüscher et al. 2014, Louarn et al. 2020, Grange et al. 2024). This difference between warm and temperate climates fits with our conceptual model, as temperate grass–legume mixtures are based on C_3_ and not C_4_ grasses, explaining the difference in optimum for both environments. In our case, and in particular for rhizoma peanut, this optimum range supports and validates the recently proposed strip-planting method (Castillo et al. 2014, Shepard et al. 2022), which aims to achieve a meaningful but far from dominant legume presence. Despite the perception that the more legume the better, targeting this realized optimum proportion will reduce the high establishment costs of this species as lesser area needs to be planted within a pasture (usually ∼50%), which could facilitate wider adoption. Further research is needed to clarify whether this optimum target of realized legume proportion differs with different N levels and age of stand.

Studying canopy attributes of the grass-only and grass–legume mixtures (Figs 2 and 3), we found that a similar LAI was needed in both canopy types to intercept the same fraction of PAR (∼4), aligning with the findings of Husse et al. (2016). These results, however, differ from the classical study of Brougham (1958), where pastures including legumes intercepted the same fPAR with lower LAI, associated with more horizontal leaves of legumes [white clover (*Trifolium repens* L.) in this case]. Our results indicate that both bahiagrass monoculture and grass–legume mixtures of very similar light coefficient extinction may approach photosynthetic light saturation at similar LAI (Sinclair and Horie 1989); thus, LAI would not be a covariate affecting RUE.

Despite the similar ‘optimum’ LAI between canopy types for intercepting PAR, mixtures showed denser canopies than bahiagrass monocultures in both studies. This information derived from linear models can also be tested with power law models, likely indicating very similar results. The fact that canopy types have similar LAI when intercepting the same fPAR but achieve the same LAI at different compressed canopy heights has implications for botanical composition and grazing management. Further, this difference in the canopy height–LAI relationship between canopy types is likely underestimated, as the compressed canopy height measured using the rising plate meter is influenced by the density of the vegetation. Our results suggest that intensive grazing (i.e. shorter post-grazing canopy height) favours legume proportion in pastures due to light competition (greater LAI and consequently RUE, at same height), as empirically reported by Spasiani et al. (2023). This advantage for the legume would be intensified under N-limited conditions because of the ability of rhizoma peanut to fix a significant amount of atmospheric N (Dubeux et al. 2017). In these conditions, it is expected that legumes will outcompete grasses for light and N, two main resources affecting botanical composition (Tilman 1985).

The lower aboveground RUEs observed in our study compared with values reported for other pastures (e.g. Ojeda et al. 2018, Druille et al. 2019, Grigera and Oesterheld 2021) are likely attributable to differences in methodology. For example, Bélanger et al. (1994) and Cristiano et al. (2015) reported that 16%–55% of the total biomass produced is ignored when assessing RUE of only the aboveground biomass accumulation. The belowground biomass could be very important in our rhizomatous pastures (Ortega-S et al. 1992), which differ from RUE studies on short-term perennial pastures (2–3 years), where plants prioritize shoot biomass allocation. Regardless of this limitation, our results were consistent across different studies and years and identified ∼30% as the optimum in legume proportion in this subtropical grassland. In addition, our analysis assumes a constant energy concentration for each g of aboveground DM produced per MJ [e.g. same digestibility (IVDOM) and crude protein (CP)]. However, increasing the proportion of legumes in pasture increases non-linearly the energy concentration of each unit of DM produced, up to a certain legume proportion (e.g. ∼45% in Kohmann et al. 2022). Hence, adjusting the RUE based on energy concentration would determine increases in the optimum legume proportion from 30% to ∼40%. Beyond this point, we do not expect the energy concentration compensates for the lower RUE, as both IVDOM and CP tend to plateau at higher legume proportions (Kohmann et al. 2022).

## Conclusion

The results from two different studies indicate that aboveground RUE is limited by N when legume proportion is below 30% in unfertilized canopies. When legume proportion exceeds 30%, a physiological limitation to RUE is imposed due to replacement of the C_4_ grass with a C_3_ legume. This suggests that within the optimum range, legume proportion overcomes the N limitation, without the need of N fertilizer, while RUE is not physiologically limited. Based on our empirical evidence, moderate legume proportion could be used as a target to maximize productive, environmental, and economic outcomes in subtropical and tropical grasslands. Further research is needed to determine whether this optimum holds across different environments and species. Nonetheless, our proposed ecophysiological conceptual model could serve as a reference for other warm-climate regions.

## Supplementary Material

plaf036_Supplementary_Data

## Data Availability

The data and R code used in this study are available in https://github.com/CaramNico/AoBP.
