# Supplementary material for: Nutritional and physiological limitations shape the radiation-use efficiency response to legume proportion in C4 grass–legume mixtures
Source: AoB Plants. 2025 Jun 26;17(4):plaf036. doi: 10.1093/aobpla/plaf036 (PMC12260219; doi:10.1093/aobpla/plaf036)
Supplement: plaf036_Supplementary_Data [file plaf036_supplementary_data.pdf]

Appendix:

Appendix 1 Study areas. The small-plot study 1 area is defined in red lines, assessed during 2021 and 2022. The grazed Study 2 area is defined in white lines, assessed during 2022 and 2023.

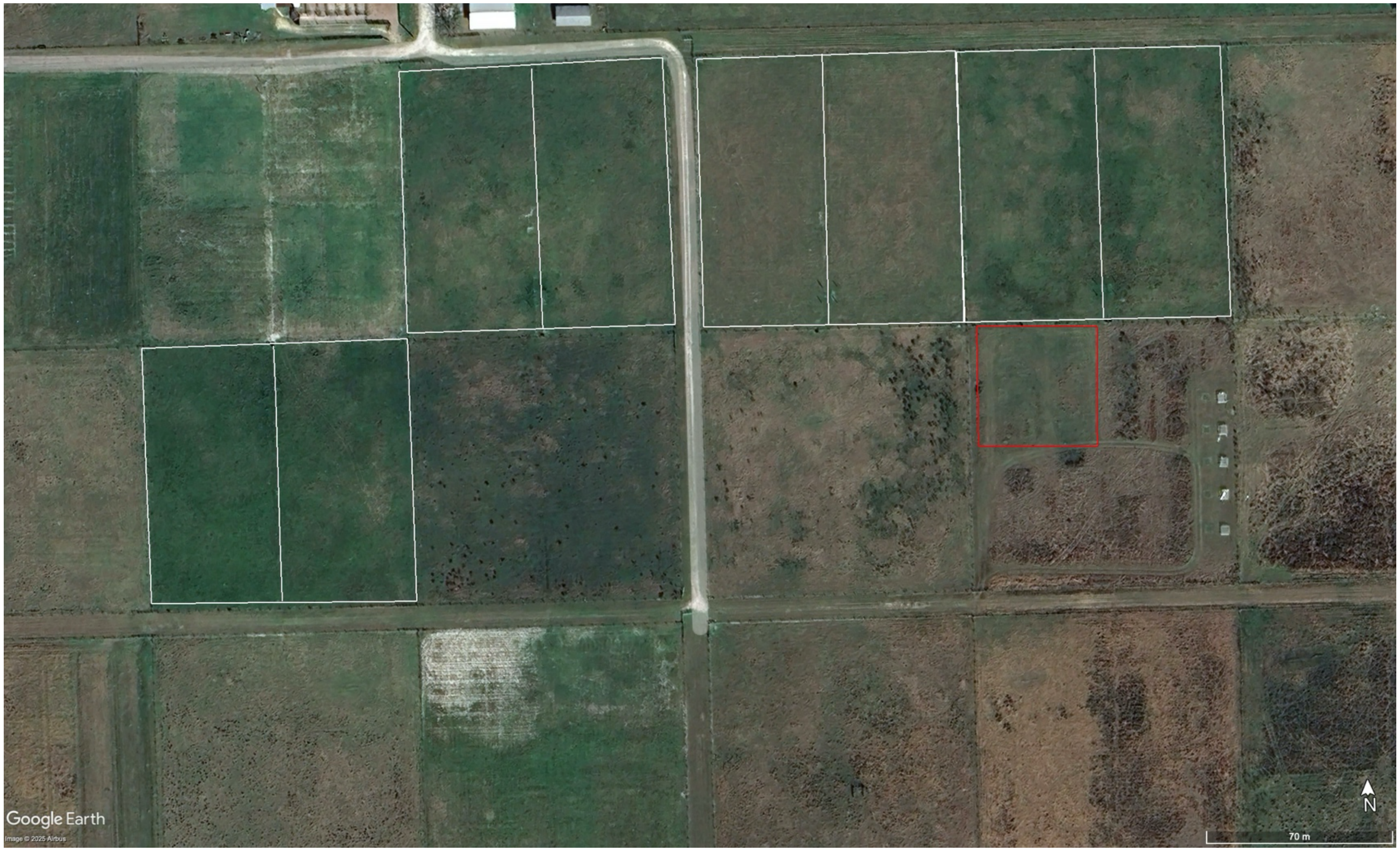

Appendix 2 Study 1 Akaike information criteria (AIC), corrected AIC (AICc) and root mean square error (RMSE) of the four candidate models for the relationship between RUE and legume proportion for the pooled data (overall response including year 1 and year 2), and for each year. Each model was fitted for the median (50th), the frontier (95th) and the limited relationship (10th). The RMSE was estimated through the leave-one-out cross-validation (LOOCV) technique.

		10th			50th			95th		
	Candidate models	AIC	AICc	RMSE	AIC	AICc	RMSE	AIC	AICc	RMSE
Overall	Linear	**43.6**	**43.6**	**0.344**	77.3	77.4	0.254	224.2	224.3	0.552
	Quadratic	45.0	45.1	0.330	70.9	71.2	0.249	204.5	204.7	0.516
	Plateau linear	44.1	44.3	0.333	**69.1**	**69.4**	**0.245**	**199.9**	**200.1**	**0.510**
	Plateau quadratic	47.1	47.4	0.330	71.3	71.6	0.246	201.4	201.6	0.504
2021	Linear	**57.9**	**58.1**	**0.322**	50.2	50.5	0.246	107.3	108.2	0.561
	Quadratic	**55.9**	**56.3**	**0.312**	**45.7**	**46.3**	**0.243**	**96.3**	**96.6**	**0.594**
	Plateau linear	60.0	60.4	0.320	**48.1**	**48.7**	**0.244**	**101.0**	**101.4**	**0.571**
	Plateau quadratic	62.3	62.9	0.317	49.9	50.8	0.245	**101.0**	**101.6**	**0.584**
2022	Linear	**-31.1**	**-31.0**	**0.237**	-1.7	-1.4	0.199	86.8	86.9	0.535
	Quadratic	-29.7	-29.4	0.237	-1.6	-1.1	0.200	74.2	74.5	0.498
	Plateau linear	-29.2	-28.9	0.243	**-3.3**	**-2.8**	**0.197**	**77.2**	**77.5**	**0.483**
	Plateau quadratic	-19.9	-19.4	0.234	-1.3	-0.6	0.198	**74.0**	**74.4**	**0.503**

Note: 'Best' candidate models are in bold according to the model metrics (lowest AIC/AICc and/or RMSE).

Appendix 3 Study 1 Akaike information criteria (AIC), corrected AIC (AICc) and root mean square error (RMSE) of the four candidate models for the relationship between RUE and legume proportion for the seasonal data for both years (late spring: May and June; early summer: July; mid-summer: August; late summer: September-October). The RMSE was estimated through the leave-one-out cross-validation (LOOCV) technique.

		Late spring			Early Summer			Mid-Summer			Late Summer		
	Candidate models	AIC	AICc	RMSE	AIC	AICc	RMSE	AIC	AICc	RMSE	AIC	AICc	RMSE
Study 1	Linear	44.1	44.7	0.294	2.8	3.6	0.188	-3.0	-2.2	0.176	-54.9	-54.3	0.109
	Quadratic	43.6	44.6	0.301	-0.7	0.8	0.191	-3.8	-2.3	0.181	**-56.6**	**-55.6**	**0.104**
	Plateau linear	**43.1**	**44.0**	**0.289**	**-0.9**	**0.6**	**0.172**	**-5.0**	**-3.6**	**0.170**	-56.2	-55.3	0.105
	Plateau quadratic	44.7	46.1	0.298	0.4	2.7	0.199	-2.1	0.2	0.186	-54.3	-52.9	0.104

Note: 'Best' candidate models are in bold according to the model metrics (lowest AIC/AICc and/or RMSE).

Appendix 4 Study 2 Akaike information criteria (AIC), corrected AIC (AICc) and root mean square error (RMSE) of the four candidate models for the relationship between RUE and legume proportion for the pooled data (overall response including year 1 and year 2), and for each year. Each model was fitted for the median (50th), the frontier (95th) and the limited relationship (10th). The RMSE was estimated through the leave-one-out cross-validation (LOOCV) technique.

		10th			50th			95th		
	Candidate models	AIC	AICc	RMSE	AIC	AICc	RMSE	AIC	AICc	RMSE
Overall	Linear	298.7	298.8	0.721	254.8	255.0	0.409	366.3	366.4	0.871
	Quadratic	**293.3**	**293.4**	**0.701**	**233.3**	**233.5**	**0.383**	**343.1**	**343.3**	**0.773**
	Plateau linear	308.6	308.9	0.772	295.0	295.1	0.428	362.1	362.2	0.876
	Plateau quadratic	355.5	355.7	0.745	297.1	297.3	0.427	369.5	369.7	0.866
2022	Linear	**128.0**	**128.1**	**0.699**	111.9	112.2	0.413	170.3	170.4	0.909
	Quadratic	128.5	128.9	0.692	**107.9**	**108.4**	**0.404**	**165.2**	**165.5**	**0.868**
	Plateau linear	147.5	147.8	0.780	136.9	137.2	0.429	172.5	172.8	0.894
	Plateau quadratic	146.9	147.2	0.762	123.9	124.5	0.434	174.5	175.0	0.906
2023	Linear	165.7	165.8	0.717	144.4	144.6	0.435	194.2	194.3	0.895
	Quadratic	**163.2**	**163.4**	**0.700**	**130.6**	**131.1**	**0.420**	**165.3**	**165.6**	**0.832**
	Plateau linear	169.1	169.4	0.715	147.6	148.0	0.443	186.1	186.4	0.919
	Plateau quadratic	162.7	163.1	0.707	147.9	148.3	0.448	210.6	211.0	0.907

Note: ‘Best’ candidate models are in bold according to the model metrics (lowest AIC/AICc and/or RMSE).

Appendix 5 Study 2 Akaike information criteria (AIC), corrected AIC (AICc) and root mean square error (RMSE) of the four candidate models for the relationship between RUE and legume proportion for the seasonal data for both years (late spring: May and June; early summer: July; mid-summer: August; late summer: September-October). The RMSE was estimated through the leave-one-out cross-validation (LOOCV) technique.

		Late spring			Early Summer			Mid-Summer			Late Summer		
	Candidate models	AIC	AICc	RMSE	AIC	AICc	RMSE	AIC	AICc	RMSE	AIC	AICc	RMSE
Study 2	Linear	45.4	45.9	0.321	37.6	38.2	0.291	38.7	39.3	0.311	**-11.5**	**-10.8**	**0.177**
	Quadratic	**40.4**	**41.4**	**0.312**	**29.8**	**30.8**	**0.269**	**33.2**	**34.3**	**0.284**	-9.6	-8.5	0.183
	Plateau linear	62.7	63.2	0.392	52.9	53.5	0.330	47.2	47.8	0.315	-2.3	-1.1	0.199
	Plateau quadratic	60.5	61.5	0.363	51.5	52.6	0.263	47.5	48.6	0.317	-5.8	-4.1	0.214

Note: 'Best' candidate models are in bold according to the model metrics (lowest AIC/AICc and/or RMSE).
